# The impact of carbon source on cell growth and the production of bioactive compounds in cell suspensions of *Hancornia speciosa* Gomes

**DOI:** 10.1038/s41598-021-03845-0

**Published:** 2021-12-21

**Authors:** Luciana Arantes Dantas, Paula Sperotto Alberto Faria, Bruno Matheus Mendes Dário, Ana Luíza Martins Arantes, Fabiano Guimarães Silva, Roniel Geraldo Avila, Paulo Sérgio Pereira, Aurélio Rubio Neto

**Affiliations:** 1 Plant Biotechnology, Program in Biotechnology and Biodiversity, Pro-Centro Oeste Network-Federal Institute of Education, Science and Technology Goiano (IF Goiano), Rio Verde, GO Brazil; 2 Plant Tissue Culture Lab, IF Goiano, Rodovia Sul Goiana, Km 01, Zona Rural, Rio Verde, GO CEP: 75.901-970 Brazil; 3 Biomolecules and Bioassays Laboratory, IF Goiano, Rio Verde, GO Brazil

**Keywords:** Biotechnology, Plant sciences

## Abstract

Belonging to the Brazilian flora, the species *Hancornia speciosa* (Gomes), known as mangabeira, has bioactive compounds of interest, such as flavonoids, xanthones, and proanthocyanidins. The objective of this study was to determine how the supplementation of sugars in culture medium affects the osmotic potential of the medium, as well as its influence on cell growth and on the concentration of phenolic compounds. For this purpose, after 90 days of subculture, 20 mL aliquots of the cultures were added to flasks containing 20 mL of medium with different sugars (glucose, fructose, sucrose, mannitol, and sorbitol) under a 16-h photoperiod with a spectral range between 400 and 700 nm of photosynthetically active radiation (45–55 μmol m^−2^ s^−1^) in a shaker at 110 rpm. After 30 days, the pH, electrical conductivity, osmotic potential, biomass accumulation, and concentrations of phenolic compounds were evaluated. Regardless of their concentration in the medium, the sugars sorbitol and mannitol provided more unfavorable conditions for water absorption at the cellular level, reducing the water potential of the medium. Sucrose favored greater water absorption and biomass accumulation. Among the various sugar concentrations, 3% (30 g/L) sucrose or glucose improved the accumulation of fresh and dry cell weight and the production of polyphenols such as chlorogenic acid, epicatechin, rosmarinic acid, hesperidin, rutin, and quercetin. In addition, they resulted in a higher osmotic potential of the medium and larger cells than other carbon sources. Despite the differences in cell size, no culture conditions compromised cell survival.

## Introduction

The plant *Hancornia speciosa* (Gomes), known as *mangabeira*, is present in tropical geoenvironments such as the Brazilian Cerrado, plateaus, and coastal lowlands. They are found in areas of open vegetation such as fields, *cerradão* (savanna forest), *campo sujo* (open savanna), dunes, and restinga forests (coastal sandy plain vegetation) and do not tolerate shade. Naturally, they predominate in areas with sandy, acidic soils that are poor in nutrients and organic matter and have low water-holding capacity. Appreciated by the local population, the fruit of the *mangabeira* stands out for its nutritional and chemical richness: it is high in pectin, vitamins C and E, iron, zinc, β-carotene, and tannic phenolic compounds. Named by the Brazilian Ministry of the Environment as the plant of the future, it has been the subject of important advances in the areas of biotechnology, processing, and medical research^1^.

Natural products have been the source of medicinal agents with various applications for many years and remain an abundant source of new chemotypes and pharmacophores, valuable bioactive agents. The commercial interest of these bioactive compounds comes from the possibility of altering the production of the plants through tissue culture technology, since several species of interest grow poorly outside their native environments^2,3^. Despite the presence of bioactive substances such as rutin and cyclitol l-(+)-bornesitol, there are no studies on the in vitro production of bioactive compounds in *H. speciosa*^4^.

On this background, plant cell culture has gained importance because it is a biotechnology topic of great pharmaceutical relevance because it allows the synthesis of phytochemicals in greater quantity in the short term, since the compounds are produced under controlled conditions independent of external factors found in nature, reducing labor costs and improving yield^5^. In such systems, undifferentiated cells are used for the production of high-value secondary metabolites in vitro*,* such as resveratrol, taxol, artemisinin, and ginsenosides^6^. This technology has numerous advantages for the production of active compounds, since it allows more reliable production and faster and more efficient isolation of the phytochemical than isolation from whole and complex plants. In addition, it allows greater control of the culture environment, the elimination of interfering compounds found in plants, and the supply of plant material with markers for use in laboratory animals, and it serves as a model for elicitation tests^5,7,8^.

Several strategies can optimize the yield of these bioactive compounds in in vitro cultures, including the composition of the medium with different concentrations and carbon sources, as it plays a key role in the genetic regulation of the signal transduction system and in the cell development process^6^. The addition of carbon sources at different concentrations can significantly influence the performance of the culture due to their impact on the energy supplied to the cell and maintenance of the osmotic potential of the medium^9^. The dysregulation of these physiological cell survival-related factors can lead to significant changes in cell expansion and division, as well as in the biosynthesis of secondary metabolites^10^. Therefore, the objective of this study was to evaluate the effects of different carbon sources and concentrations on the modulation of cell growth and the production of bioactive compounds in *H. speciosa* cells cultured in a cell suspension.

## Material and methods

### Plant material

Fruits of different *mangabeira* plants (*H. speciosa*) from the Gameleira Farm in the georeferenced region of the municipality of Montes Claros de Goiás, state of Goiás (GO), Brazil (16° 0′ 28″ S, 51° 23′ 49″ W) were transported to the Plant Tissue Culture Laboratory of the Goiano Federal Institute, Rio Verde Campus, GO. The collection of biological material complied with legal aspects in accordance with federal law No. 12.651 of May 25, 2012. Considering the provisions of Article 21, the collection of fruits in small volumes fulfilled the ripening period, and in accordance with the mentioned guidelines, for the study it was not necessary institutional authorization to carry out the collection.

After manual pulping, the seeds were coated with gauze and immersed in running water for 30 min, followed by 70% alcohol for 1 min, and then immersed in sodium hypochlorite (NaClO) solution (20% of the commercial solution) with a drop of polysorbate (Tween^®^) for 15 min. Then three rinses were performed in a laminar flow hood using autoclaved distilled water to remove residues of the disinfecting solutions.

**Figure 1:**
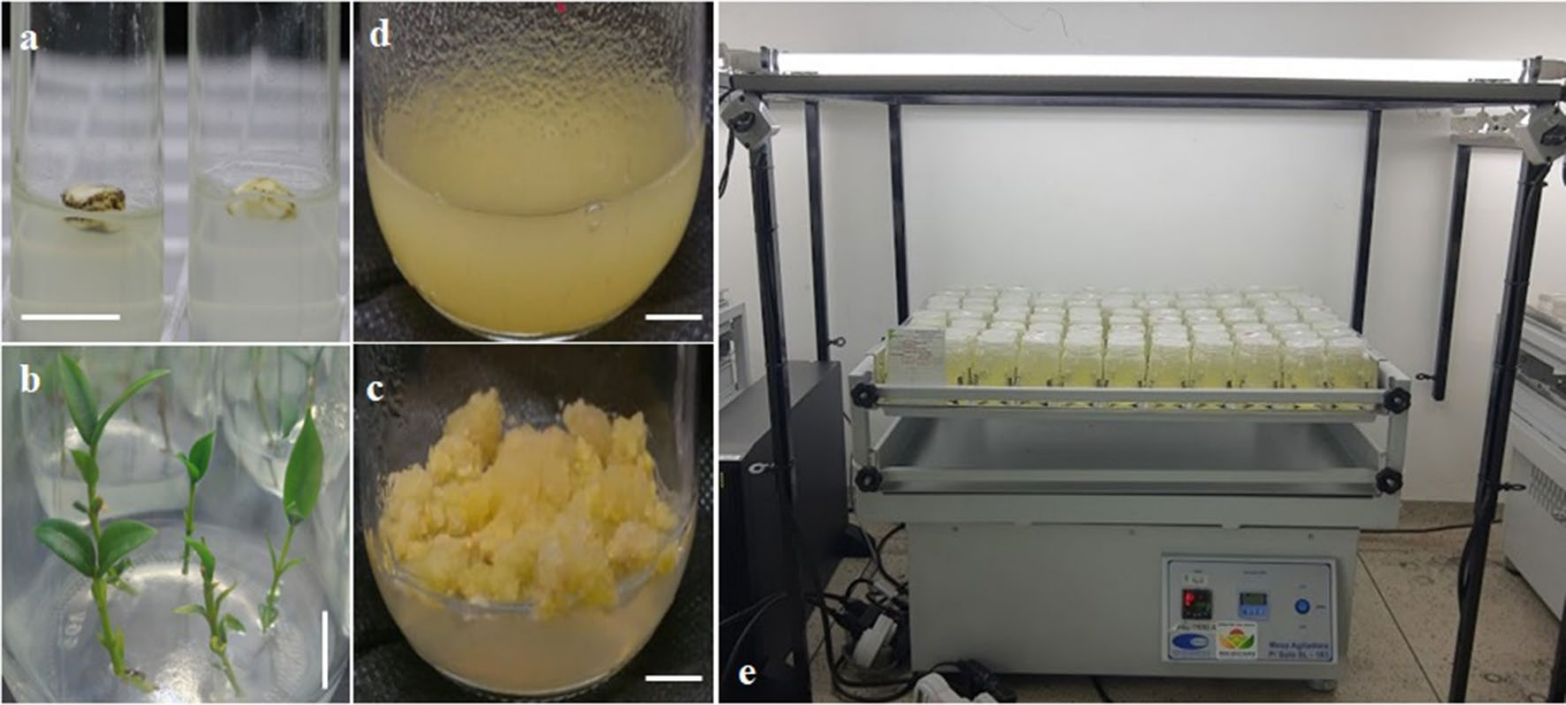
Process of the in vitro establishment and culture of *H. speciosa* (Gomes) cell culture. Seeds disinfected and inoculated in a tube (**a**). Explants obtained after 60 days of culture (**b**). Friable calli 120 days after the beginning of induction from leaves (**c**). Stable cell suspension (**d**). Orbital shaker used to culture the cell suspension under light support with 50 μmol m^−2^ s^−1^ irradiance (**e**). Bar = 1 cm.

Disinfected seeds were inoculated aseptically in test tubes (25 × 150 mm) containing 10 mL of MS medium^11^, with 50% concentrations of salts and vitamins, 30 g L^−1^ of sucrose, 3.5 g L^−1^ of agar (Dinâmica^®^), and pH adjusted to 5.7 ± 0.3 (Fig. 1a). The medium had been autoclaved at 121 °C and 1.05 kg cm^−2^ pressure for 20 min. The test tubes were sealed with a plastic (polypropylene) lid and kept in a growth room at a temperature of 25 ± 3 °C and relative humidity of 45 ± 2%, and the medium was changed every 30 days. They were kept under a photoperiod of 16 h under photosynthetically active radiation (45–55 μmol m^−2^ s^−1^) provided by 20-W LED lamps (Lanao Tubes series, China) whose spectral composition (400–700 nm) was verified using a USB2000 spectroradiometer (OceanOptics). Explants pre-established in vitro (Fig. 1b) were kept under these conditions.

### Callus induction and cell suspension

Leaf fragments (1 cm^2^) of previously established seedlings were inoculated in glass flasks containing 40 mL of 50% MS medium and 30 g L^−1^ of sucrose supplemented with 3.5 g L^−1^ of agar. To the growth medium was added a combination of growth regulators at a concentration of 2.5 mg L^−1^ of naphthalene acetic acid (NAA) and 1 mg L^−1^ of 6-benzylaminopurine (BAP), and the pH was adjusted to 5.7 ± 0.3. The flasks containing the cultures were kept in a room with the same environmental conditions under which the explants were obtained.

To establish the cell-suspension culture, friable calli (Fig. 1c) were selected and transferred to 250-mL glass flasks containing 40 mL of medium with the respective concentrations of salts and growth regulators in which the calli had been cultured but without agar. The cultures were placed under agitation (shaker LS-183, Solab™) at 110 rpm under the same light and temperature conditions described above (Fig. 1d,e). After 3 months, stable and contamination-free cultures were selected to begin the elicitation process.

### Carbon source in the culture medium

To initiate the elicitation process, approximately 20 mL of inoculum and 20 mL of 50% MS medium containing 1 mg L^−1^ BAP and 2.5 mg L^−1^ NAA were added, supplemented with different carbon sources (sucrose, glucose, fructose, sorbitol, and mannitol) at different concentrations (1, 2, 3, 4, and 5%) and with the pH adjusted to 5.7 ± 0.03 before autoclaving. The flasks were kept on an orbital shaker at 110 rpm for 30 days in a growth room under the same environmental conditions mentioned above.

### Evaluations

#### Biometric characteristics

At the end of the culture period after the addition of eliciting agents, the pH, electrical conductivity, and osmotic potential were measured in the suspensions, which were then filtered through 0.45-µm filter paper to remove the culture medium. After filtration, the fresh cell weight was measured, and the samples were then placed in a forced-air oven at 35 °C ± 2 for 24 h to measure the dry weight.

#### Electrical conductivity, pH, and osmotic potential

A portable conductivity meter and pH meter were used to measure the electrical conductivity and pH of the cell suspensions, respectively. The Vapro^®^ osmometer (model 5600) was used to evaluate the osmotic potential.

#### Cell viability and cell area

For cell viability analysis, a 50-µL aliquot was added to the cell-suspension culture sample in a 1.5-mL microtube along with 50 µL of 0.2% trypan blue dye (Sigma-Aldrich, Brazil). Next, 50-µL aliquots were transferred to slides and counted under an optical microscope (BX61, Olympus). The plant cells were photomicrographed using a U-photo system with a DP73 camera. These images were analyzed using ImageJ^®^ software, version 1.52, to measure the cell areas.

#### Phytochemical analysis

The extracts were prepared using 0.1 g of dry sample with 2 mL of methanol-HPLC (Neon) in an ultrasound bath for 30 min. The extracts were filtered through cotton and a membrane filter (Advantec HP020AN—20 µm). Next, the chromatographic analysis was performed in a Shimadzu HPLC with a SPD-M20A photodiode detector (λ = 254 nm) and an LC18 column (25 cm × 4.6 mm, 5 µm, Supelcosil) coupled to a 2-cm LC18 precolumn (Supelguard, Supelco) in an oven set at 30 °C.

Ten microliters of each extract was injected in triplicate, and the phenolic compounds were detected. The elution flow rate was set to 1 mL/min, with 0.1% acetic acid as mobile phase A and HPLC-grade methanol (Neon) as mobile phase B. The concentration of mobile phase B increased from 10 to 66% over 32 min, decreased from 66 to 10% from 32 to 35 min, and stayed at 10% for 5 min, for a total run time of 40 min.

The compounds present in the samples were detected by comparison with the peaks of known phenolic and flavonoid standards, and quantification was performed using the standard-curve equations for the following standards: gallic acid, epicatechin, caffeic acid, chlorogenic acid, ferulic acid, orientin, vitexin, rosmarinic acid, myricitrin, isovitexin, hesperidin, rutin, quercetin-3-glucoside, kaempferol-3-galactoside, quercitrin, kaempferol-3-glucoside, kaempferol-3-rutinoside, quercetin, kaempferol, luteolin, and apigenin.

#### Statistical analysis

The experimental design was completely randomized, in a 5 × 5 factorial arrangement, with five carbon sources (sucrose, glucose, fructose, sorbitol, and mannitol) at five different concentrations (1, 2, 3, 4, and 5%), with five replicates (40-mL flask). After analysis of variance with the *F* test (5%), the means of the quantitative variables were analyzed by regression and the means of the qualitative factors by Tukey’s test. These tests were run in SISVAR software at 5% probability^12^.

Pearson’s correlation was used to quantify the relationships between the variables analyzed. To obtain the estimates, the software R version 3.5.2 (R Development Core Team, 2018) was used, and the heatmap graph was prepared using the Corrplot package.

## Results

### Growth profile and cell behavior

**Figure 2:**
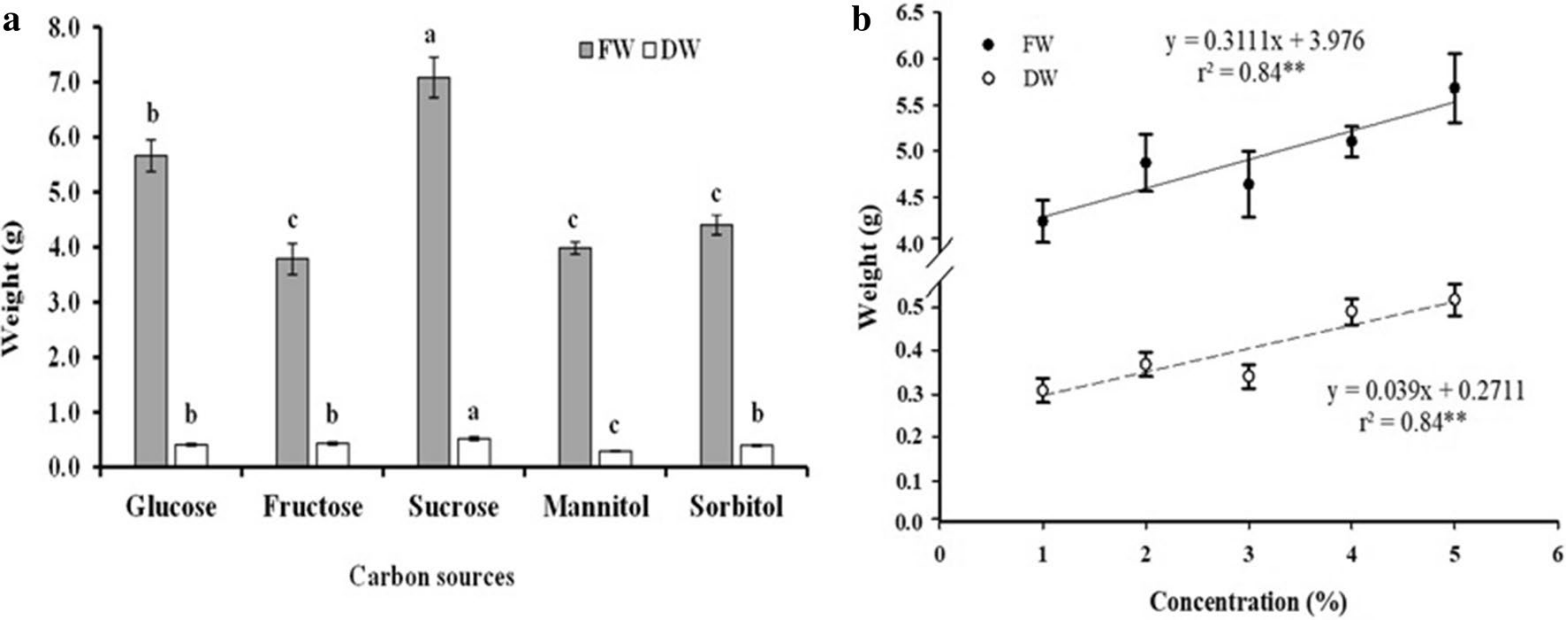
Accumulation of fresh and dry weight of cell suspension cultures of *H. speciosa* (Gomes) grown at different concentrations and carbon sources. The bars indicate the standard error of the mean; significance: **p < 0.01.

The highest fresh cell weight was obtained in the presence of sucrose, followed by glucose, while sorbitol, mannitol, and fructose resulted in lower fresh weights. Regarding dry weight accumulation, sucrose remained prominent, but mannitol provided the lowest accumulation (Fig. 2a). Observing in isolation the factor of concentration in which the cultures were subjected, it is noted that regardless of the carbon source, the fresh and dry weight increased proportionally with the increase in concentration (Fig. 2b).

#### Cell viability and cell area

**Figure 3:**
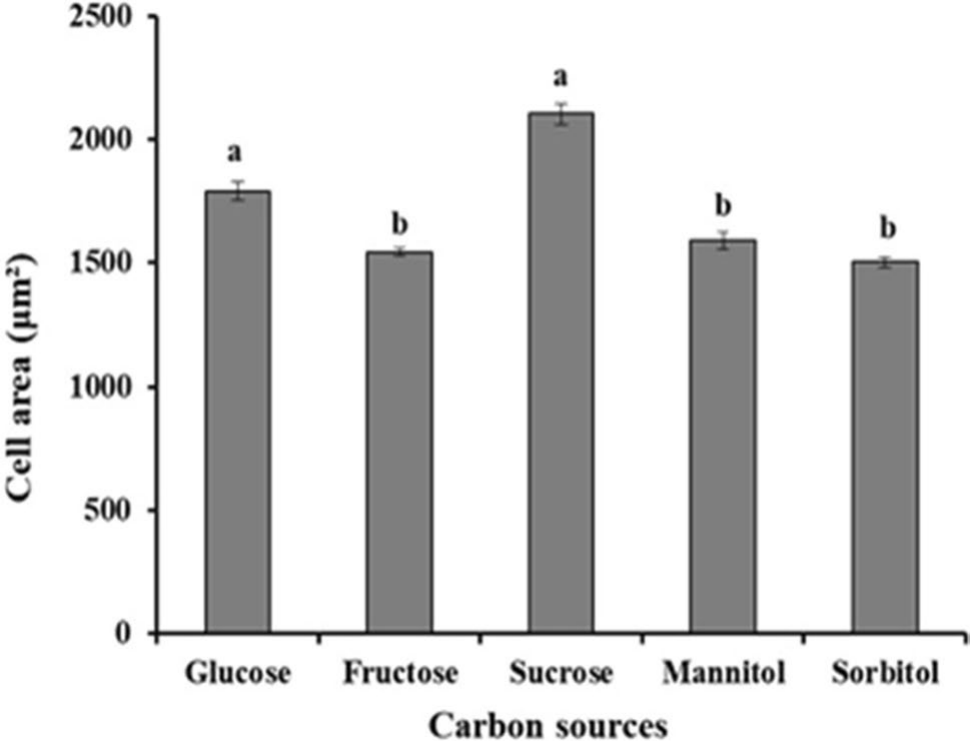
Cell areas (μm^−2^) observed in cell suspension cultures of *H. speciosa* (Gomes) in different carbon sources. Means were compared using the Tukey test. The bars indicate the standard error of the mean; significance: **p < 0.01.

**Figure 4:**
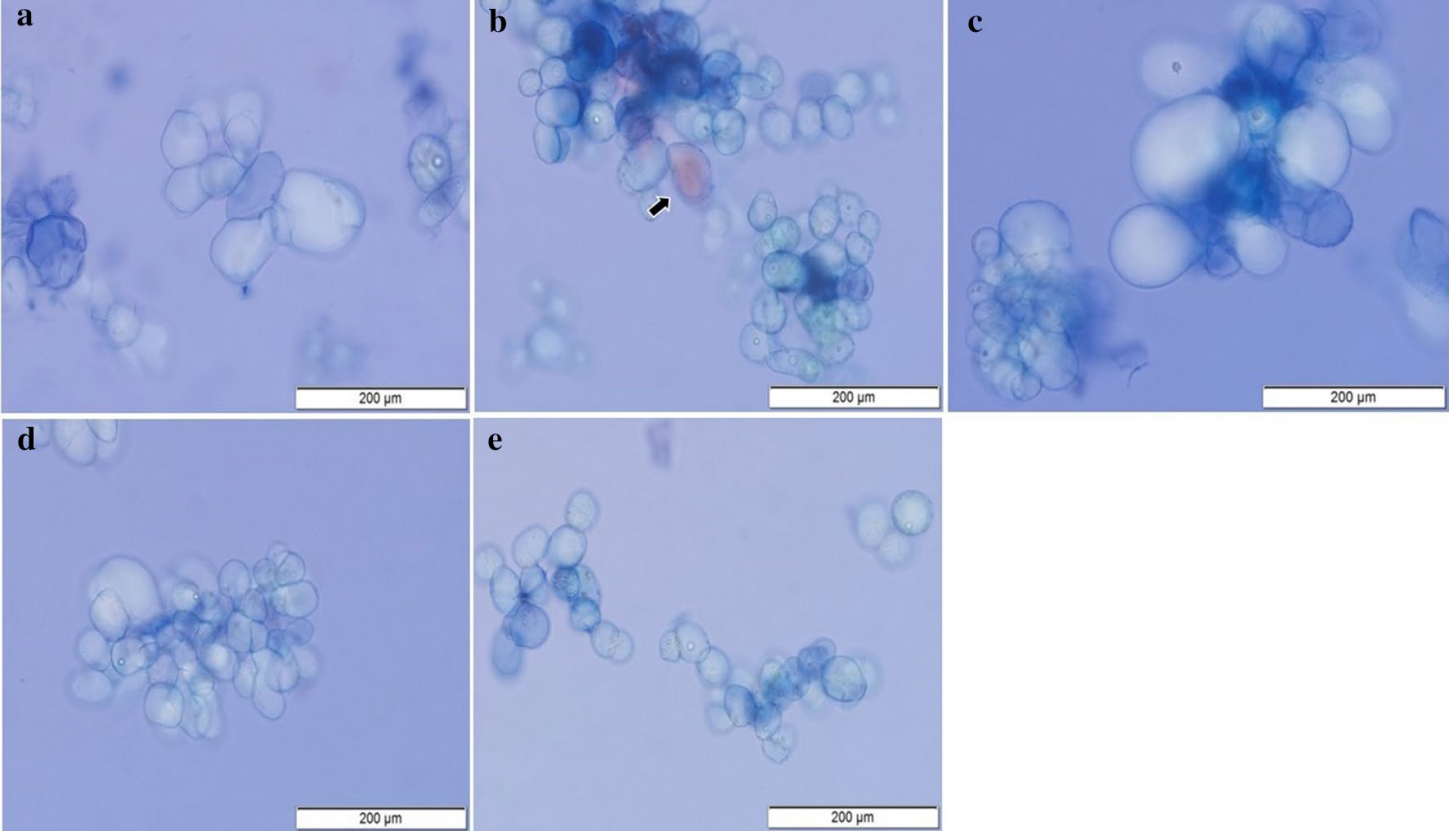
Photomicrographs of *H. speciosa* (Gomes) cells grown under different carbon sources; glucose (**a**); fructose (**b**); sucrose (**c**); mannitol (**d**); sorbitol (**e**); Bar = 200 μm. The means were compared by Tukey’s test. The bars indicate the standard error of the mean; significance: **p < 0.01.

Examining the area of the cells cultured in different carbon sources, sucrose (2102 μm^−2^ ± 43.7) and glucose (1790 μm^−2^ ± 36.6) were the most favorable conditions for obtaining larger cells (Fig. 3). The cells were smallest when cultured with fructose (Fig. 4b) mannitol (Fig. 4d) and sorbitol (Fig. 4e). Despite the different cell sizes obtained under the different the culture conditions, cell survival was not compromised, always staying above 94% ± 2 live cells.

The culture medium supplemented with sucrose or glucose provided larger cells than the other sugars tested (Fig. 4a–c). When we compared the results under different concentrations of sugars, cells cultured in 3% (2981 μm^−2^ ± 0.06) or 5% (2267 μm^−2^ ± 0.19) sucrose were larger due to the better water status of the medium. Cells cultured in the presence of 1% or 2% fructose produced pigments suggestive of anthocyanins, which are glycosylated flavonoids that help protect the cell against free radicals produced under stress (Fig. 4b).

#### Electrical conductivity and potential of hydrogen

**Figure 5:**
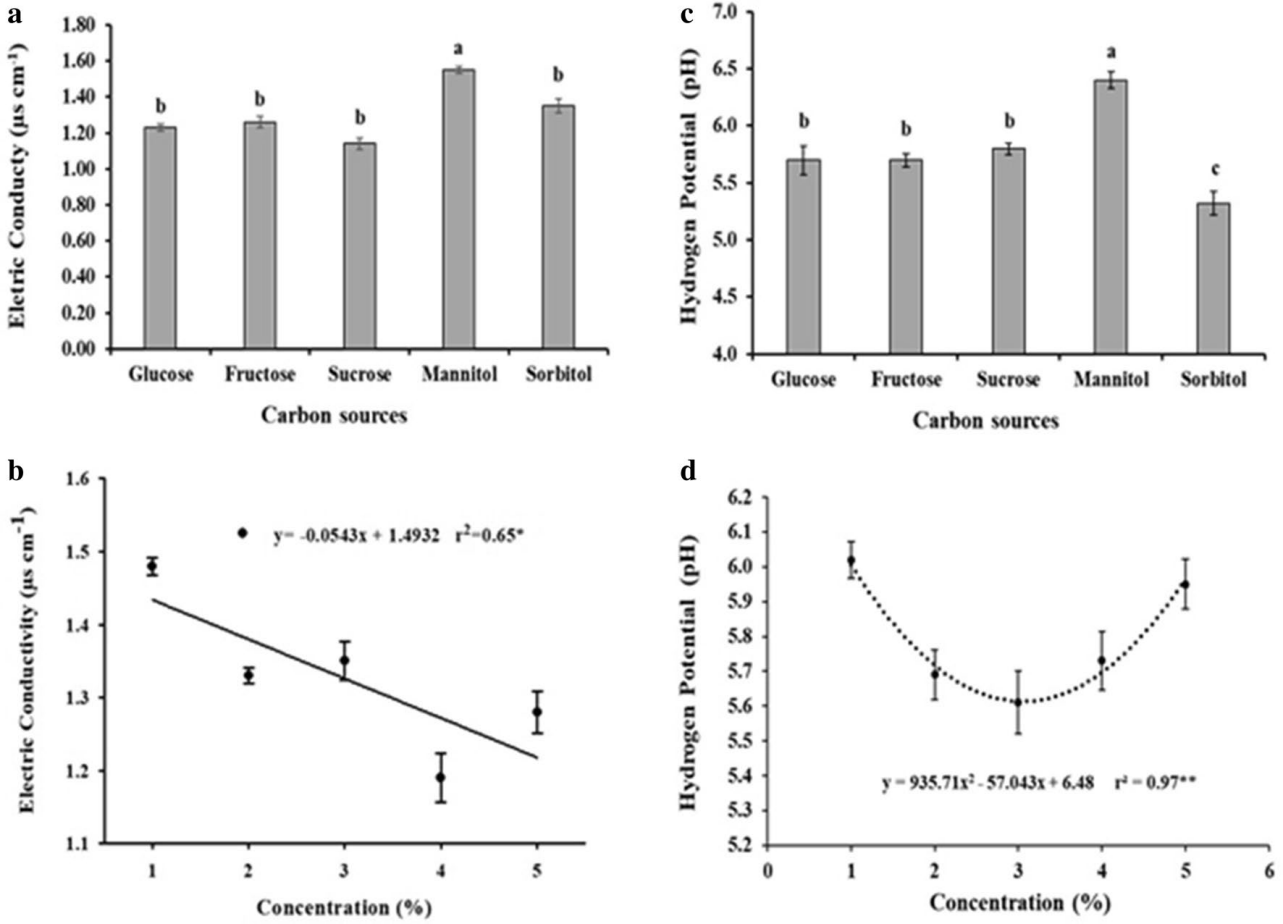
Electrical conductivity (**a**,**b**) and pH (**c**,**d**) of *H. speciosa* (Gomes) cell-suspension cultures supplemented with different concentrations of carbon sources. The means were compared by Tukey’s test. The bars indicate the standard error of the mean; significance: *p < 0.05; **p < 0.01.

The cell-suspension cultures cultured with mannitol exhibited the highest values of electrical conductivity (Fig. 5a). This fact may explain their worse accumulation of cell weight, since their absorption of free ions from the medium was compromised. Nevertheless, under the other conditions, electrical conductivity decreased as the concentration of sugar in the medium increased. Despite this, in the other conditions, decay occurred as the concentration in the medium increases (Fig. 5b).

Cell-suspension cultures supplemented with mannitol showed the highest medium pH, while sorbitol cultures had the lowest pH (Fig. 5c). Regardless of the carbon source, evaluating only the concentration factor, the lowest pH of the medium was observed at a concentration of 3%, while 1 and 5% had the highest pH (Fig. 5d).

### Osmotic potential

**Figure 6:**
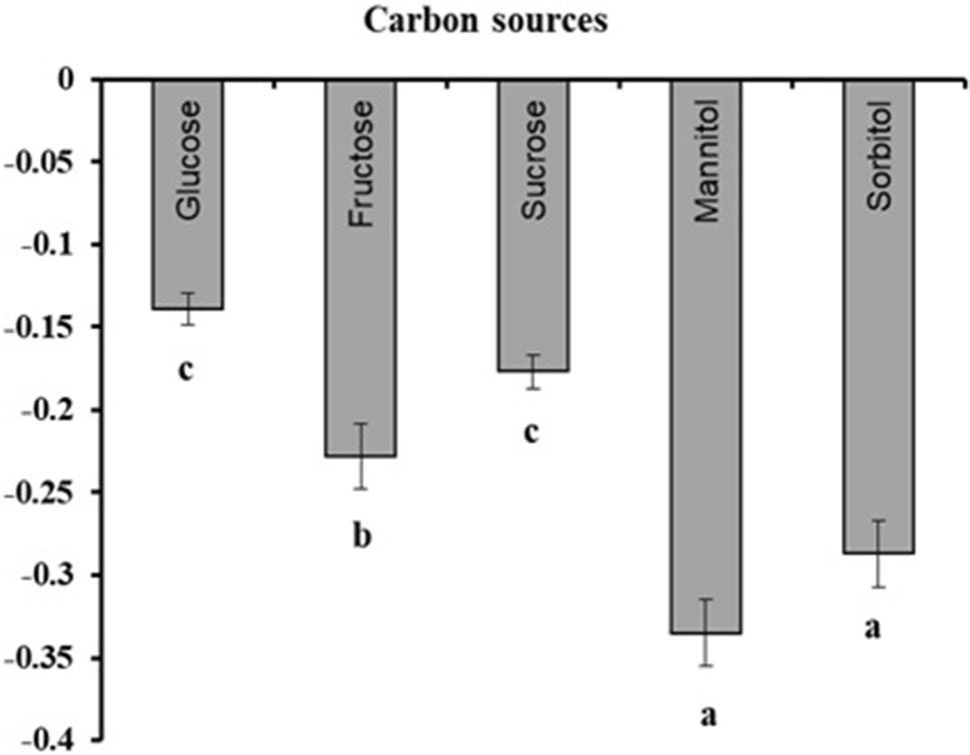
Osmotic potential (MPa) of cell-suspension cultures of *H. speciosa* (Gomes) supplemented with different carbon sources (glucose, fructose, sucrose, mannitol, and sorbitol). The means were compared by Tukey’s test. The bars indicate the standard error of the mean; significance: **p < 0.01.

The culture media containing sorbitol (− 0.287 MPa ± 0.02) and mannitol (− 0.335 MPa ± 0.02) had the lowest osmotic potential, followed by fructose (− 0.228 MPa ± 0.02). Media containing sucrose (− 0.177 MPa ± 0.01) and glucose (− 0.139 MPa ± 0.01) did not differ and resulted in the highest potential in the medium (Fig. 6).

**Figure 7:**
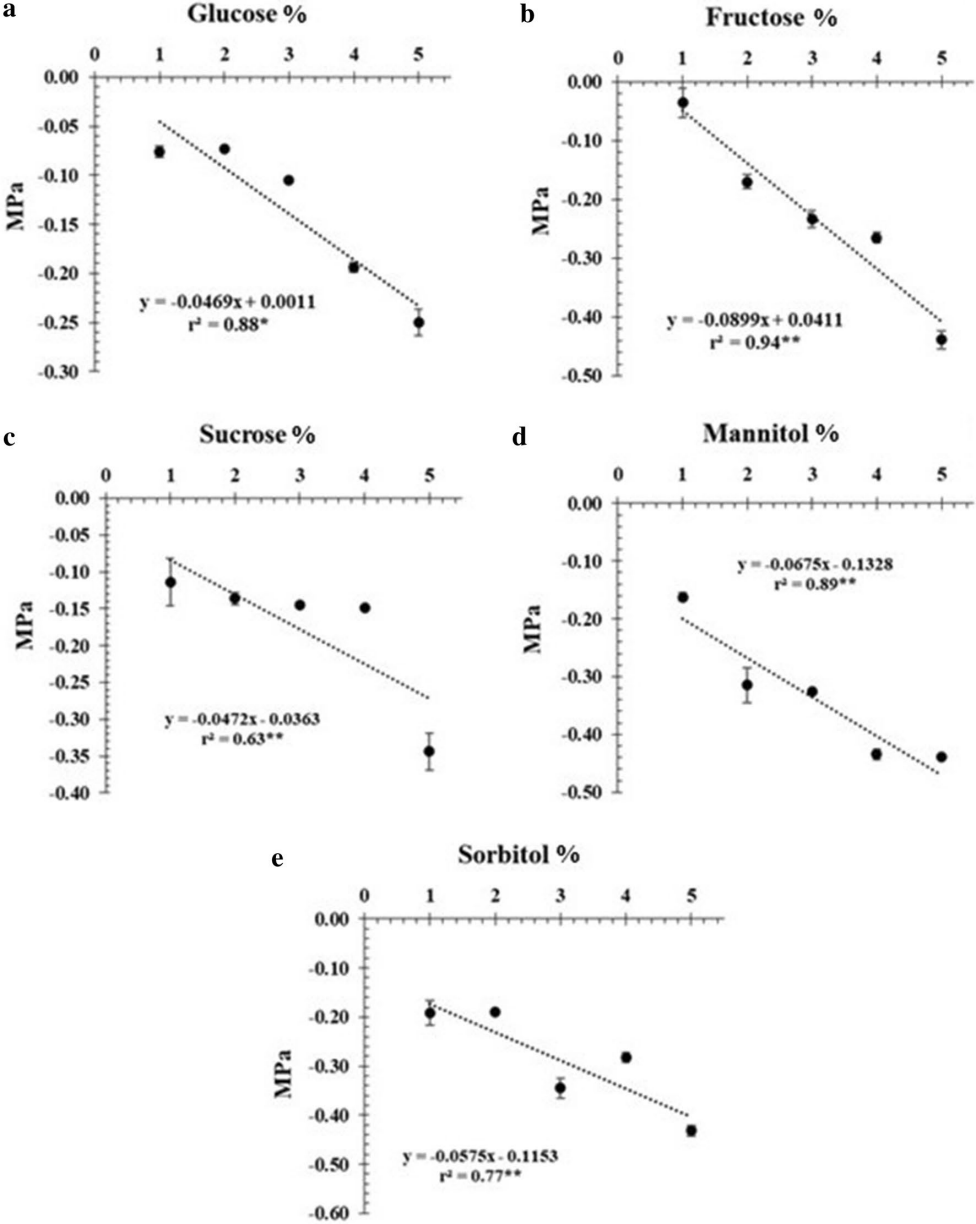
Osmotic potential (MPa) of cell-suspension cultures of *H. speciosa* (Gomes) supplemented with different sources and carbon concentrations. The bars indicate the standard error of the mean; significance: *p < 0.05; **p < 0.01.

When evaluating the different concentrations, we observed that as the sugar content in the culture medium increased, the osmotic potential decreased (Fig. 7a–e). Regardless of the concentration in the medium, the sugars sorbitol (Fig. 7e) and mannitol (Fig. 7d) provided more unfavorable conditions for water absorption and consequently nutrient absorption at the cellular level, reducing the water potential of the medium. In contrast, sucrose favored greater water absorption and weight accumulation. Therefore, sucrose provided a better osmotic potential of the medium, favoring the growth of *H. speciosa* cells in suspension (Fig. 7c).

### Detection and quantification of bioactive compounds

**Figure 8:**
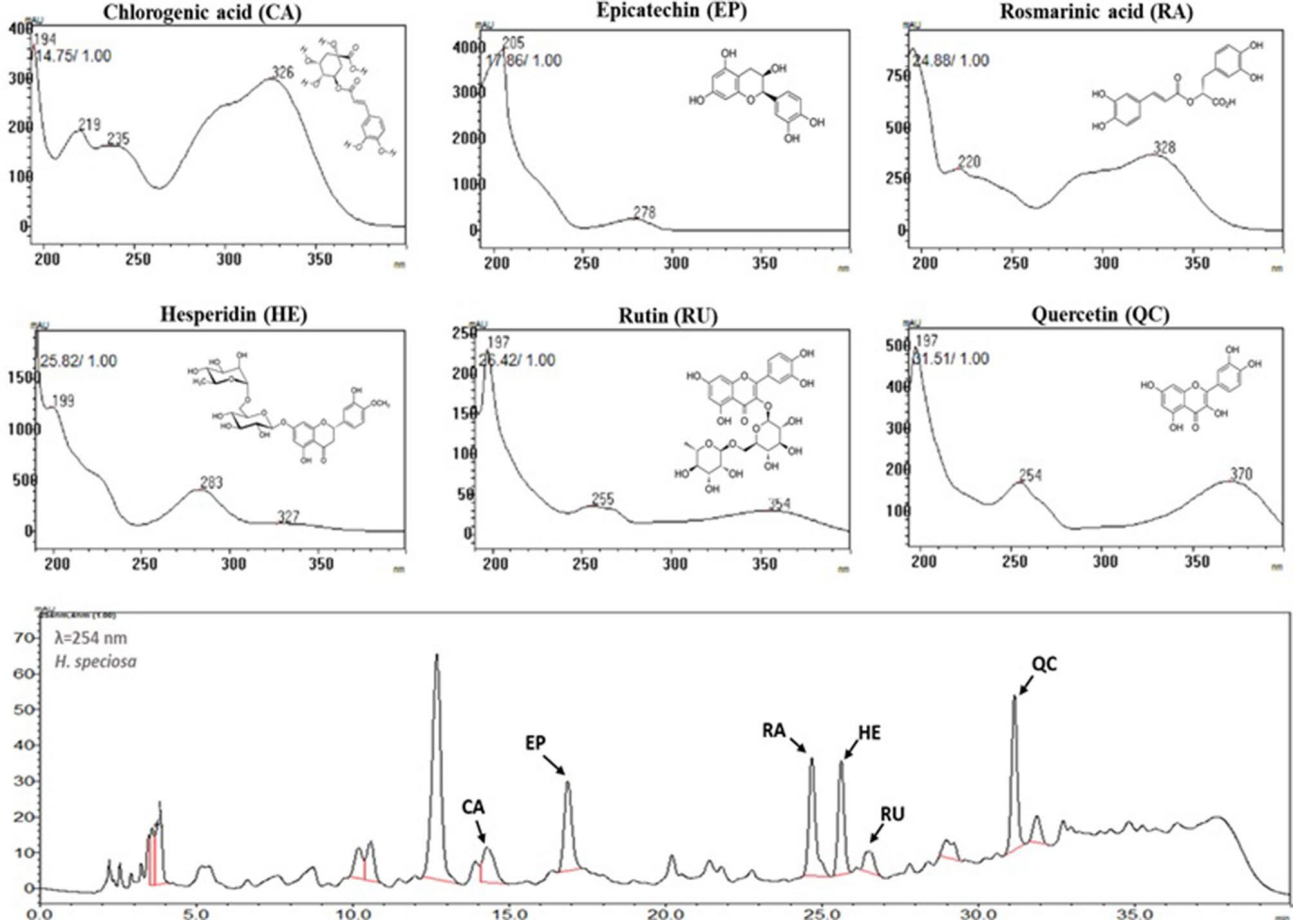
Spectra and chromatographic separation of phenolic compounds obtained by HPLC-DAD from *H. speciosa* (Gomes) cell suspensions grown with different carbon sources.

Under the different culture conditions, the retention times (RTs) of the following compounds were detected: chlorogenic acid (RT: 14.75), epicatechin (RT: 17.86), rosmarinic acid (RT: 24.88), hesperidin (RT: 25.82), rutin (RT: 26.42), and quercetin (RT: 31.51) (Fig. 8).

**Figure 9:**
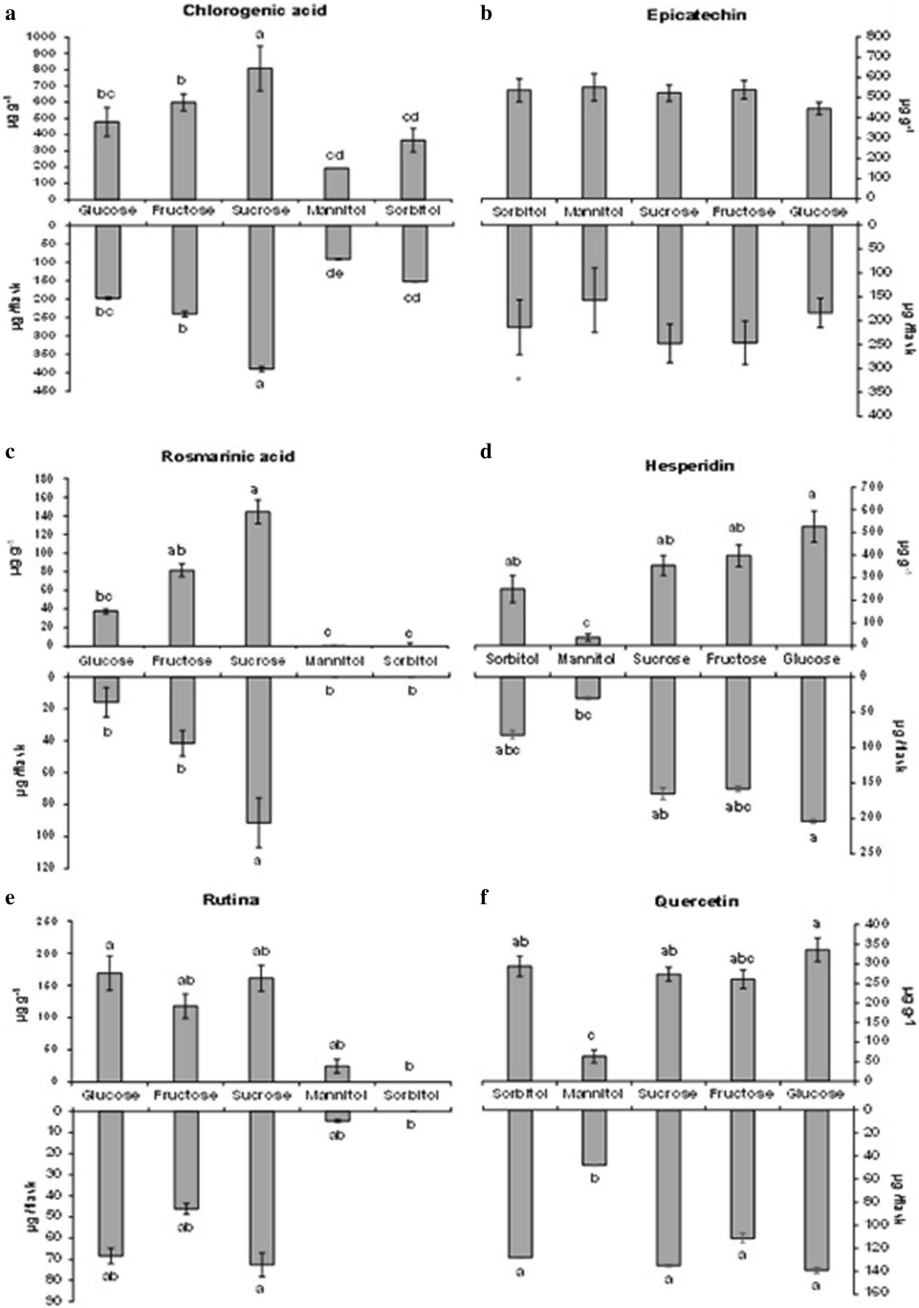
Concentration and yield of chlorogenic acid (**a**), epicatechin (**b**), rosmarinic acid (**c**), hesperidin (**d**), rutin (**e**), and quercetin (**f**) in cell-suspension cultures of *H. speciosa* (Gomes) supplemented with different carbon sources. The means were compared by Tukey’s test. The bars indicate the standard error of the mean; *not significant.

**Figure 10:**
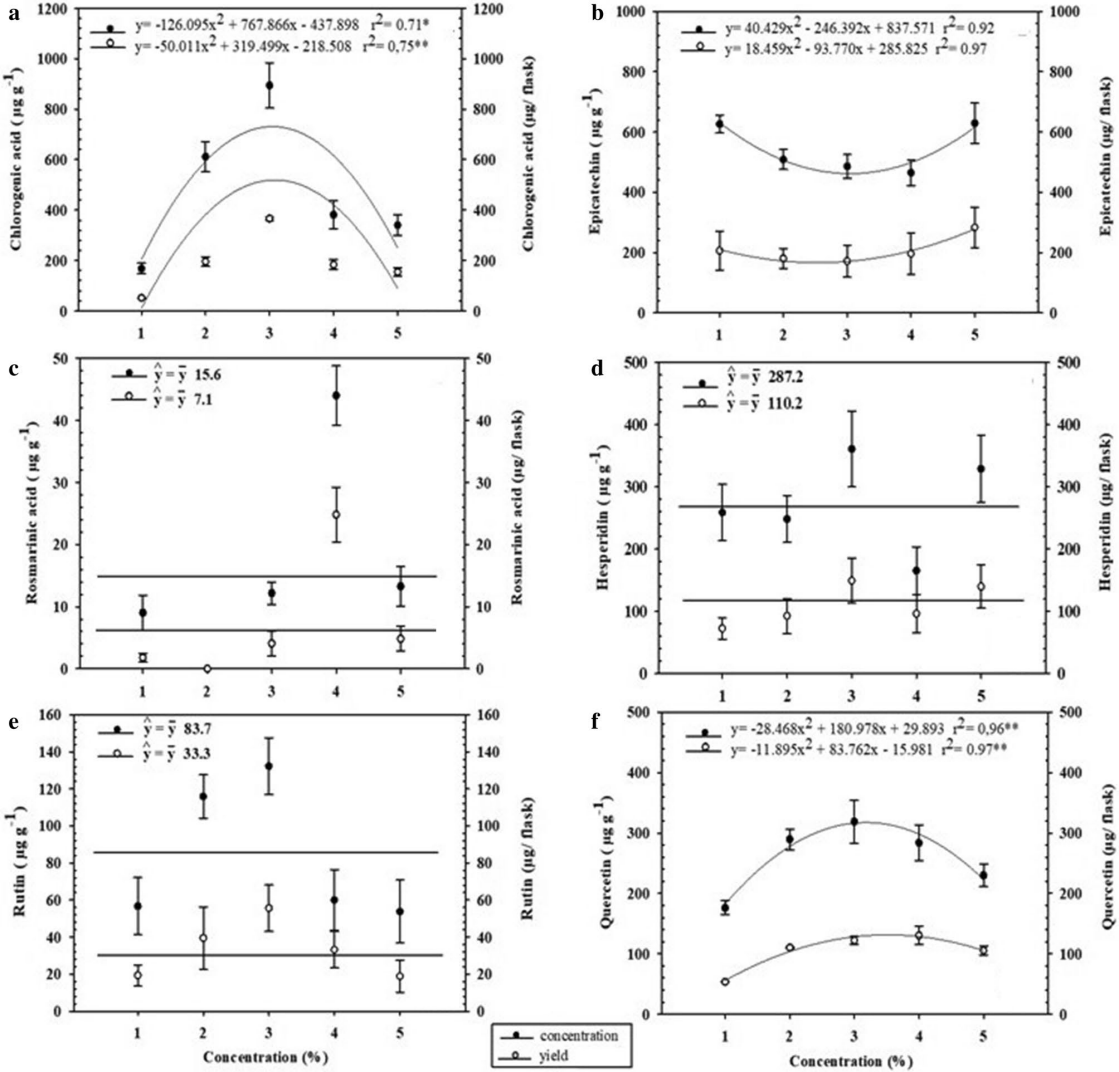
Concentration and yield of chlorogenic acid (**a**), epicatechin (**b**), rosmarinic acid (**c**), hesperidin (**d**), rutin (**e**) and quercetin (**f**) of the cell-suspension cultures of *H. speciosa* (Gomes) supplemented with different concentrations. The bars indicate the standard error of the mean.

The concentration and yield of different metabolites in the analyzed samples differed by carbon source. Chlorogenic acid was produced more highly by cultures supplemented with 3% sucrose than the other culture conditions (Figs. 9a and 10a), in terms of both concentration (808 μg g^−1^ ± 138) and yield (389 μg/flask ± 6.8).

Epicatechin was produced similarly between the carbon sources, with an average concentration of 543 μg g^−1^ ± 48.7 and 207 μg ± 3.4 yield per flask (Figs. 9 and 10b).

Rosmarinic acid production was produced at the highest level under 4% sucrose (Fig. 9c) (Fig. 10c), with a maximum concentration of 144 μg g^−1^ ± 43.2 and yield of 91.3 μg/flask ± 27.3.

Hesperidin production was produced the least in suspensions cultured with mannitol and the most in suspensions with glucose (concentration: 526 μg g^−1^; yield: 204 μg/flask) (Fig. 9d). The 3% concentrations were the best for producing hesperidin (Fig. 10d).

 Glucose proved to be the best carbon source for producing the highest concentration of rutin (169.3 μg g^−1^ ± 26.6), but when evaluating the yield, sucrose achieved the highest value (72.5 μg/flask ± 5.6) (Fig. 9e). Sorbitol inhibited rutin production, which was not detected at any of the concentrations of sorbitol tested. Among the other supplementation sources, the concentration of 3% achieved the highest concentration (132.2 μg g^−1^ ± 25.3) and yield (55.6 μg/flask ± 12) (Fig. 10e).

 Mannitol was the carbon source with the lowest quercetin production (63.1 μg g^−1^ ± 16.4), whereas cultures supplemented with glucose produced approximately 432% more (335.7 μg g^−1^ ± 29.9) than this concentration (Fig. 9f). When evaluating the yield of this metabolite, mannitol (47.9 μg/flask ± 0.06) was unfavorable for the accumulation of quercetin. Supplementation with glucose (139.1 μg/flask ± 2.34), fructose (111.4 μg/flask ± 4.7), sucrose (135 μg/flask ± 0.99), or sorbitol (128 μg/flask ± 0.16) favored the yield, and these did not differ from each other. Considering the concentrations of carbon sources in the media, all behaved quadratically, with the highest values observed with the supplementation of 3% of all carbon sources tested (Fig. 10f).

**Figure 11:**
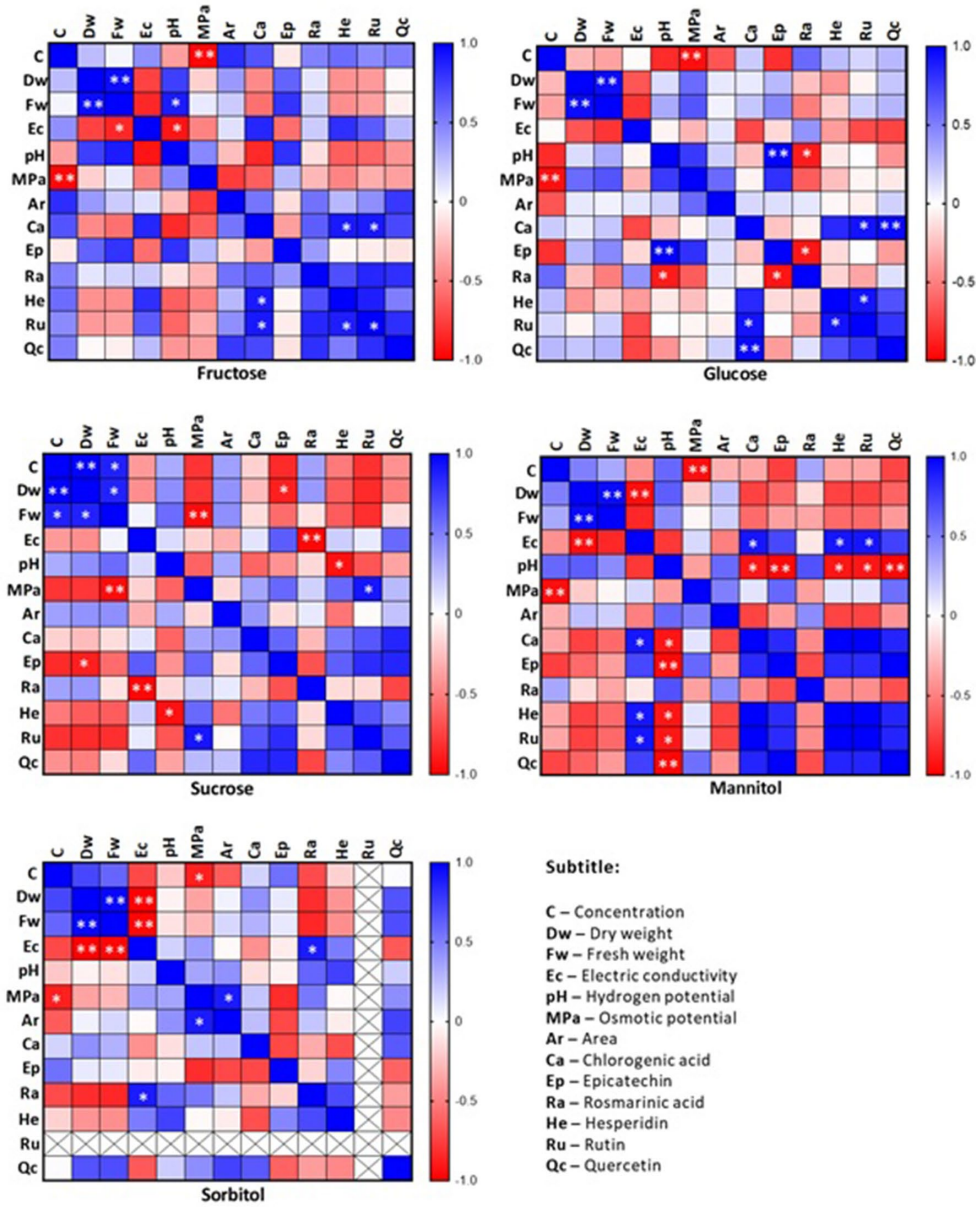
Estimates of heatmap correlations according to Pearson’s coefficient for multiple variables (dry weight; fresh weight; pH; electrical conductivity; osmotic potential; cell area; chlorogenic acid, epicatechin, rosmarinic acid, hesperidin, rutin, and quercetin concentrations) after 30 days of *H. speciosa* (Gomes) cell culture in different carbon sources. ** and * are significant at 1 and 5% by the *t* test, respectively. Significance: *p ≤ 0.05, **p ≤ 0.01.

 When studying the association between the variables analyzed through their correlation coefficients, in the culture conditions with fructose or glucose, a positive correlation was observed between the production of chlorogenic acid and the concentrations of hesperidin, rutin, and quercetin (Fig. 11). The osmotic potential was not correlated with the production of phenolic compounds detected in this study, except for the production of rutin when the cells were cultured in sucrose: the higher the potential, the greater the amount of rutin in the sucrose cultures.

Cells cultured in medium supplemented with mannitol showed evidence of increased pH and conductivity, which caused lower production of chlorogenic acid, epicatechin, hesperidin, rutin, and quercetin. This confirmed, together with the above data, that a lower the production of these compounds came with a higher pH of the medium.

## Discussion

The carbon source and its concentration in in vitro culture medium directly modulate the process of clonal multiplication of *Chonemorpha fragrans* Alston seedlings^13^. This is due to the influence of the carbon source on cell division and differentiation, as energy plays a crucial role in the progression through the different phases of the plant cell cycle^14^. During the suspension culture of cells, the carbon source can be an extremely important variable for the success of the technique in terms of the cellular multiplication of seedlings in vitro.

In fact, in this study, the carbon source strongly influenced cell growth. The best sources of carbon for the accumulation of fresh and dry weight of cells occurred in cell suspensions supplemented with sucrose, followed by glucose. This result is partly related to the maintenance of cell growth that these carbon sources allowed, given that the cell area when the energy source was sucrose or glucose was higher than when another carbon source was used. However, it is important to note that despite having the same cellular area, the cell suspensions supplemented with sucrose provided greater dry weight than those supplemented with glucose, indicating that this carbon source influences the biosynthesis of reserves and cellular constituents.

In plants, sucrose is the main transport carbohydrate and is directly involved in cell signaling^15,16^. In cell suspension, a process similar to that observed in seeds may occur because there are no differentiated tissues, and energy must be obtained from the culture medium. Thus, most of the sucrose absorption by the cells possibly results from the activity of the cell wall acid invertases, which promotes sucrose cleavage, generating hexoses, glucose, and fructose, which can generate sugar signals or be allocated for energy production during cell growth^17,18^.

In this study, the fresh and dry weights exhibited different behaviors, especially in cultures supplemented with glucose. Supplementation with sucrose or glucose provided higher accumulation of fresh weight. However, when observing the dry weight, sucrose still stood out because glucose performed similarly to fructose and sorbitol, indicating that cultures with glucose induced the cell to absorb a large amount of water, leading it to expand. In addition, in presence of the breakdown of sucrose, glucose contributes more to cell turgor than fructose.

Most likely, the cell-suspension cultures with glucose and sucrose showed better performance due to the rate of absorption of these sugars from the medium because these treatments led to higher osmotic potential of the medium. This is to be expected since, when the sugar in the medium is increased, it linearly reduces the osmotic potential, so the reverse should also be expected. Regarding the nutrient absorption rate and weight accumulation, this behavior is also observed because the treatments with the highest dry weight were those with the lowest electrical conductivity, which represents the number of ions present in the medium^19^.

Sucrose played a significant role in the progression of cell growth and therefore favored the greater accumulation of fresh and dry cell weight. In fact, sucrose is the most abundant carbohydrate present in the phloem sap of several plants; in addition to its easy availability and low cost, it is the most commonly used carbon source in tissue culture^20^. In some assays done in cell cultures^21,22^. This carbohydrate induced higher fresh and dry weight production than other sugars, corroborating the results found in this study. Sucrose has been frequently used as a carbon source in plant tissue culture for energy supply and for induction of osmotic stress, but its optimal concentration required for each stage of in vitro culture depends mainly on the plant species^20,23^. The essential signaling pathway for the plant to reorganize in the face of energy stress was demonstrated in Arabidopsis: because the plant cell perceives high or low energy availability from the amount of sugar present, this allows the cell to set its circadian clock, reprogramming itself to perform photosynthesis throughout the day^24^.

The cellular imbalance caused by several external factors, such as nutritional and osmotic factors, can trigger metabolic processes related to oxygen, and it will thus generate oxidizing compounds as a result of these stresses. This defense system has the function of inhibiting or reducing the harm caused by these compounds in two ways, enzymatic or nonenzymatic action^25–27^. The damage caused by these injuries affects cellular structures such as lipids, proteins, and DNA, and when the morphological responses trigger interference in biosynthetic pathways, they lead to the production of antioxidant compounds such as polyphenols^28,29^. These compounds have received considerable attention for their ability to mitigate the effects of oxidative stress, as demonstrated by the prevention of tumors in rats by rosmarinic acid, a compound present in the *H. speciosa* fruit^27^. In plant species, flavonoids are the main group of phenolic compounds, occur as glycosides in vacuoles of the plant cell, and signal the imbalance caused by excess free radicals, and they are highly important in industrial production^29^. In dicotyledonous plants, flavonoids are often the only metabolites with pharmacological activity^30^.

As a technology that can facilitate industrial processes, suspension cultures are a “green” biotechnological approach that represent a valuable source of active ingredients. In addition to being free of pathogens, pollutants, and agrochemical residues, which can contaminate most plant-derived extracts, plant tissue cultures rarely contain toxic compounds or potential allergens^31,32^. Several studies using this technology have aimed at optimizing and enabling the production of active compounds in bioreactors. In this study, with *H. speciosa* cells, it was shown that different sugars in the medium could modify the production of chlorogenic acid, rosmarinic acid, epicatechin, hesperidin, rutin, and quercetin.

In cell culture of *Prunella vulgaris* L.^33^, the authors found variations in the biomass and production of secondary metabolites as the sucrose concentration changed, in which high concentrations negatively influenced the production of antioxidants, as observed in the present study with *H*. *speciosa* cells. This effect is due to the high osmotic pressure inhibiting the biosynthesis of these compounds. Other authors found that the sucrose level affected the accumulation of carotenoids by *Ajuga multiflora* Bunge in in vitro cultures^34^. The anthocyanin content in *Ajuga reptans* (bugle) cell suspension was higher in medium containing sucrose concentrations up to 3% than those with concentrations above 3%^35,36^. In *Artemisia annua* L. cell suspensions, the artemisinin content was also higher under these conditions^37^. We observed the same in *H. speciosa* cells, which produced higher concentrations of phenolic compounds with 3% sucrose, confirming that biosynthesis is regulated by light and sugar.

Cultures supplemented with mannitol inhibited the production of bioactive compounds, and the pH was more alkaline than it was with the other sugars. These effects can be analyzed in future studies to elucidate the effect of the H^+^ electrochemical potential on the maintenance of the membrane under these conditions. In *Prunella vulgaris* L., an acidic medium was the condition with the highest phenolic production and biomass accumulation by this species^33^.

This study detected polyphenol production (chlorogenic acid, epicatechin, rosmarinic acid, hesperidin, rutin, and quercetin) by cells in suspension. These are compounds with potential or proven worth in the treatment of poor circulation and various skin disorders, including signs of aging, diseases, and lesions. These compounds have significant potential to inhibit or even reverse the signs of aging, such as wrinkles or hyperpigmentation marks, making them promising molecules for the development of new cosmetic formulations^26^.

There are few reports of using Brazilian Cerrado plants as biofactories, especially the native species *H. speciosa*, which highlights the great biotechnological advancement of this study. This species is perennial and is difficult to propagate but one of the most promising for sustainable exploitation programs and reforestation programs in degraded areas. The present study shows that *H. speciosa* cell-suspension culture is a good option for the production of phytochemicals for pharmaceutical applications and is a potential candidate for future biotechnological studies and for optimization of the production of bioactive compounds.

## Conclusion

The osmotic stress induced by different carbon sources at different concentrations was monitored by measuring the pH, electrical conductivity, and osmotic potential of the culture suspensions. We observed the biomass of *H. speciosa* and its accumulation of desirable secondary metabolites. Among the various concentrations of sugars analyzed, 3% (30 g L^−1^) sucrose and glucose improved the accumulation of fresh and dry cell weight and the production of polyphenols. In addition, they resulted in the highest osmotic potential of the medium and in larger cells than the other carbon sources. Despite the differences in cell size, no culture conditions compromised cell survival.

This study will help in the optimization of cell cultures for greater production of phytochemical-derived metabolites desired by the pharmaceutical industry. We hope that the identification and subsequent quantification of bioactive compounds in this study will be valuable to the scientific community and will provide a new, environmentally friendly alternative for the biotechnological exploitation of Brazilian Cerrado species.

## Abbreviations

MS: Murashige and Skoog
MPa: Osmotic potential
NAA: Naphthalene acetic acid
BAP: 6-Benzylaminopurine
